# The effect of Ashtanga-Vinyasa Yoga method on air force pilots’ operational performance

**DOI:** 10.3389/fpubh.2024.1334880

**Published:** 2024-05-01

**Authors:** Sara Santos, Filipe Melo, Orlando Fernandes, José Alberto Parraca

**Affiliations:** ^1^Departamento de Desporto e Saúde, Escola de Saúde e Desenvolvimento Humano, Universidade de Évora, Évora, Portugal; ^2^Comprehensive Health Research Centre (CHRC), Universidade de Évora, Évora, Portugal; ^3^Universidade de Lisboa Faculdade de Motricidade Humana: Cruz Quebrada, Lisboa, Portugal

**Keywords:** yoga, airforce pilots, operational performance, military health, aerospacial medicine

## Abstract

**Introduction:**

In today’s military landscape, optimizing performance and bolstering physical health and mental resilience are critical objectives. Introducing a 12-week Ashtanga Vinyasa Yoga Supta Method (AVYSM) to the training protocol of military trained Airforce pilots, we aim to assesses the feasibility and impact of the method.

**Materials and equipment:**

Borg Scale assesses the intensity level of physical activity during the intervention. Flight simulator data gauges operational performance responses. Postural control responses are measured using a force platform, stress responses are monitored via heart monitor, and handgrip dynamometry will measure strength. Respiratory capacity is assessed using a spirometer, body composition is evaluated using impedance balance, and aviation-related questionnaires are administered before and after the intervention period.

**Methods:**

In a randomized controlled trial, the totality of pilots from the “Masters in Military Aeronautics: aviator pilot specialist” course at the Portuguese Air Force Academy (PAA) were randomly assigned to the yoga intervention or the waiting list control groups, with participants providing written informed consent. The control group followed protocolized course classes for 12 weeks, while the intervention group integrated two weekly one-hour yoga sessions into their course.

**Results:**

The PAA has approved the implementation of this intervention protocol at Airbase 11 in Beja, highlighting its significance for the organization’s policy makers. We hypothesize that this method will enhance operational performance and, subsequently, elevate flight safety.

**Discussion:**

This research’s potential extends beyond the PAA, as it can be adapted for use in Airforce departments of other nations and various military contexts.

**Clinical trial registration:**

Évora University research ethics committee—approval number 21050. Study registered on ClinicalTrials.gov under identifier NCT05821270, registered on April 19, 2023.

## 1 Introduction

Air Force pilots, being healthy adults in a specialized profession, need specific training to maintain optimal performance and safety in the challenging conditions of aviation (1). This profession can negatively affect their performance, physical, and mental well-being, due to:

- Exposure to environmental elements includes factors such as noise, vibration, psychophysiological fatigue, and spatial disorientation (1).
- The evolution of aircraft design has brought about notable improvements in engine performance and aerodynamics, enabling pilots to navigate at elevated altitudes and contend with increased gravitational forces (G-forces). These advancements significantly amplify pilots’ psychophysiological responses, especially when combined with the effects of hypoxia, thereby markedly influencing the visual and auditory reaction times of the crew (1, 2).
- Hypoxia stands as the primary hazard during high-altitude flights, leading to increased perceived stress and exertion, elevated heart rate, and reduced functionality of respiratory muscles. Exposure to hypoxia can impair working memory and pattern recognition, posing significant challenges to pilots (3).
- Elevated physiological responses to task-related or cognitive stressors can hinder the ability to navigate and address stressful situations effectively (4).
- Demands of piloting a military aircraft include the efficient operation of the vestibular system to maintain balance within the aircraft cabin (5). Fighter pilots often show a heightened susceptibility to spatial disorientation, which can be attributed to the unique characteristics of the aircraft they pilot. Each aircraft is designed for specific functions, contributing to individual variations in susceptibility (6). An essential attribute of the postural control system is its capacity to devalue unreliable sensory information while prioritizing more dependable inputs—commonly referred to as sensory recalibration. This ability implies that, when confronted with visual impairments, reliance on alternative sensory information systems may be intensified. However, this heightened reliance requires specific training. Targeted balance training has shown promise in significantly improving postural stability by strengthening or recalibrating the remaining sensory systems (7).

Understanding these modern military demands can provide insights for specific training to better prepare pilots and aircrews in countering these threats. Recognizing these factors opens new avenues for training and cognitive preparation (3, 4).

Among various exercise options, incorporating yoga into pilot training is a practical choice for the pilots’ demands: its multisensory approach engages compensatory sensory systems in the vestibular system that improve balance, stability, and coordination, while also addressing aspects such as breathing to develop the respiratory system, physical postures to enhance flexibility and strength, embodiment for increased proprioception, and meditation for cognitive preparation (7–11). This method not only addresses the distinctive challenges encountered by pilots but also the expected enhancements in overall well-being and cognitive performance are poised to positively impact decision-making processes and operational performance during aircraft operations (4).

Yoga encompasses physical, mental, and spiritual practices originating from India. Ashtanga Vinyasa Yoga, known for its substantial movement and complex postures, is a physically demanding style of yoga. It involves interconnected physical postures (asanas), flowing movements (vinyasas), and synchronized breathing techniques (pranayama). Ashtanga emphasizes the harmony of posture, breath control, and gaze. It requires a focus on physical embodiment, coordinating each movement with inhalation or exhalation, which makes it a form of “moving meditation” (8, 9). Various yoga modalities have been shown to have predominantly positive outcomes in different populations (10). The viniyoga principle emphasizes ongoing adaptation for therapeutic benefits, covering poses, conscious breathing, meditation, and philosophy, allowing practitioners to tailor their practice to their health, age, occupation, and lifestyle (11).

In line with this methodology, a specialized yoga practice, AVYSM, tailored for Air Force Pilots has been developed. Understanding the previously mentioned challenges and responsibilities faced by Airforce pilots will be instrumental in guiding the development and evaluation of strategies and training programs, especially:

- establishing the efficacy of AVYSM in refining stress responses in individuals, thus improving cognitive response, and decision-making.
- scrutinizing the impact of AVYSM in stimulating the recalibration of the vestibular system, above all during rotational tasks implying changes in head position in space, to augment postural stability and balance, thereby mitigating spatial disorientation.
- evaluating the extent to which AVYSM contributes to enhanced overall health, body composition, strength, and respiratory performance, serving as a potential countermeasure against hypoxia threats and increased G-forces in the aircraft.

This study aims to determine the suitability of incorporating this method into the training program for Portuguese Air Force pilots, with the goal of enhancing their capabilities and increasing flight safety by preventing human error.

## 2 Materials and equipment

Material and equipment selection for the study prioritized availability, capability to measure physiological functions impacted by piloting military aircraft, suitability for real-life training integration, and efficiency in delivering results without hindrance to pilots’ daily workload, as well as adherence to the highly specific military regulations in force.

Both primary and secondary outcomes were selected following a comprehensive review of the literature and a comparison with pilots’ perceived difficulties (as identified through a brief interview, detailed in the study flow). Primary outcomes were deemed the most critical factors in flights, with Operative Performance being the most crucial for pilots’ safety. Secondary outcomes were also identified as significant contributors.

### 2.1 Primary outcomes

Changes in Operative performance—Flight times to complete tasks in the flight simulator; number of errors during the emergency protocol and their classification report from the flight simulator controller (12). The assessment will determine if the exercise protocol influences the pilot’s decision-making, as evidenced by improved task efficiency, reduced errors, and enhanced visual and auditory reaction times (1, 2)—Time Frame: measured at 12 weeks (vs. baseline values).

Changes in Stress and HRV—Heart rate variability, measured with Polar H10 portable device (2, 13–15). The assessment will reveal if the exercise protocol influences pilots’ responses to stressors, with a lower stress response enhancing decision-making (4)—Time Frame: measured at 12 weeks (vs. baseline values).

Changes in Vestibular system control responses—The Center of Pressure (CoP) represents the dynamics of the neuromusculoskeletal system, was measured with a portable force plate (Biosignals Plux-Portugal) with a sample frequency at 1000 hz. and data downsampled to 100 Hz (16, 17). Preprocessing performed using the EEGLAB toolbox, which is available for use in MATLAB (The MathWorks Inc., Natick, MA) (18). Enhanced postural control responses may indicate the protocol’s potential to mitigate the effects of spatial disorientation (6, 7)—Time Frame: measured at 12 weeks (vs. baseline values).

### 2.2 Secondary outcomes

Changes in Strength—Handgrip strength measured with a handgrip dynamometer Baseline Smedley, Model 12–0286, White Plains, NY, United States (19). The assessment will determine if the exercise protocol impacts pilot strength, crucial for maintaining control, resisting fatigue, and ensuring safety during high-intensity flights (2)—Time Frame: measured at 12 weeks (vs. baseline values).

Changes in Body composition—Body composition data (percent body fat, percent fat free mass, percent muscle mass index, as a percentages) measured with a Tanita (MC-780 MA, Tanita, Tokyo, Japan) to get a bioelectrical impedance analysis (BIA) will indicate if the exercise protocol had any impact in the pilot’s physical makeup. Percent body fat, percent fat-free mass, and percent muscle mass index collectively constitute body composition measures. Percent body fat reflects the fat proportion in total body weight, encompassing essential and storage fat. In contrast, percent fat-free mass represents the non-fat components, including muscle, bone, organs, and other tissues. Percent muscle mass index specifically gauges the proportion of body weight attributed to muscle. The relationship between percent body fat and percent fat-free mass is complementary, totaling 100%, while percent muscle mass index indicates the proportion of muscle within total body weight (20, 21). Enhanced body composition could mitigate adverse effects of the pilot profession on physical well-being (1)—Time Frame: measured at 12 weeks (vs. baseline values).

Changes in well-being and general health—measured through the SF-36 V1 questionnaire (22, 23) will indicate if the exercise protocol had any impact in the pilot’s general health subjective assessment, mitigating adverse effects of their profession on pilots’ physical well-being (1)—Time Frame: measured at 12 weeks (vs. baseline values).

Changes in Lung capacity—Ventilatory response measured with spirometry—FEV1/FVC (Forced Expiratory Volume in 1 s and Forced Vital Capacity) ratio indicates how much air you can forcefully exhale (2, 5) will indicate if the exercise protocol had any impact on lung capacity, which might offset hypoxia threats on pilots (3) – Time Frame: measured at 12 weeks (vs. baseline values).

Five Facet Mindfulness Questionnaire (FFMQ)—Changes in cognitive abilities with 15 questions and average scores are calculated by summing the responses and dividing by the number of items, and indicate the average level of agreement with each subscale (1 = rarely true, 5 = always true). Higher scores are indicative of someone who is more mindful in their everyday life (24, 25). Determining the impact of the exercise protocol on cognitive abilities, a more mindful pilot is anticipated to exhibit improved decision-making skills during work (1, 3)—Time Frame: measured at 12 weeks (vs. baseline values).

Multidimensional Assessment of Interoceptive Awareness Questionnaire (MAIA)—Changes in cognitive abilities—is an 8-scale state–trait questionnaire with 32 items to measure multiple dimensions of interoception; scores are between 0 and 5, where higher score equates to more awareness of bodily sensation (26). The evaluation will assess the exercise protocol’s impact on cognitive abilities, expecting improved decision-making in pilots with heightened awareness of bodily sensations (1, 3)—Time Frame: measured at 12 weeks (vs. baseline values).

Aviation Safety Attitude Scale (ASAS)—Changes in cognitive abilities—consists of 27 items on a five-point scale, each designed specifically to assess pilots’ attitudes with respect to predict the hazardous event involvement of aviators; For all the attitude subscales, higher scores indicate a greater degree of that particular attitude—For example, higher scores on the ASAS self-confidence factor indicated that the person expressed greater confidence in their ability as a pilot (27). The evaluation will assess the exercise protocol’s impact on cognitive abilities, anticipating enhanced decision-making skills in more confident pilots (1, 3)—Time Frame: measured at 12 weeks (vs. baseline values).

## 3 Methods

### 3.1 Study design, ethics approval and informed consent publication

The study was approved by the Évora University Research Ethics Committee, with approval number 21050, and participants gave written informed consent. The study is in agreement with the Declaration of Helsinki. The trial was registered in ClinicalTrials.gov with identifier NCT05821270, registered on 19th April 2023. Furthermore, this intervention protocol was accepted by the PAto be applied on Airbase 11 in Beja. A randomized, prospective, controlled two-arm trial will be adopted, in which one arm will be the control group (waiting list) and the other the intervention group (yoga).

### 3.2 Participants

#### 3.2.1 Sample size determination

Among all military personnel in the Portuguese Air Force, the inclusion criteria for participation in this study comprise healthy individuals serving as military pilots actively engaged in their training (tirocinium) at the PAA, which consists of taking the course titled “Masters in Military Aeronautics: Aviator Pilot Specialist.”

Conversely, the exclusion criteria encompass military pilots on active duty either preceding (not aviation pilots yet) or following the tirocinium (distributed by different airbases inside and outside the country and flying different types of aircrafts in restricted, classified or warzones), those with injuries, and individuals undergoing sudden lifestyle changes (e.g., initiating or discontinuing smoking habits, commencing, or ceasing any form of medication, or adopting a new diet).

There are currently 19 pilots actively undergoing their training (tirocinium) at the PAA, serving within “Esquadra 101 - RONCOS,” situated at Airbase 11 in Beja, which comprises the totality of this population for the PA. These pilots are divided into two classes, and they share common aircrafts, specifically the Aerospatiale Epsilon TB-30. Additionally, both classes are assigned comparable workloads and operational tasks.

**IMAGE 1:**
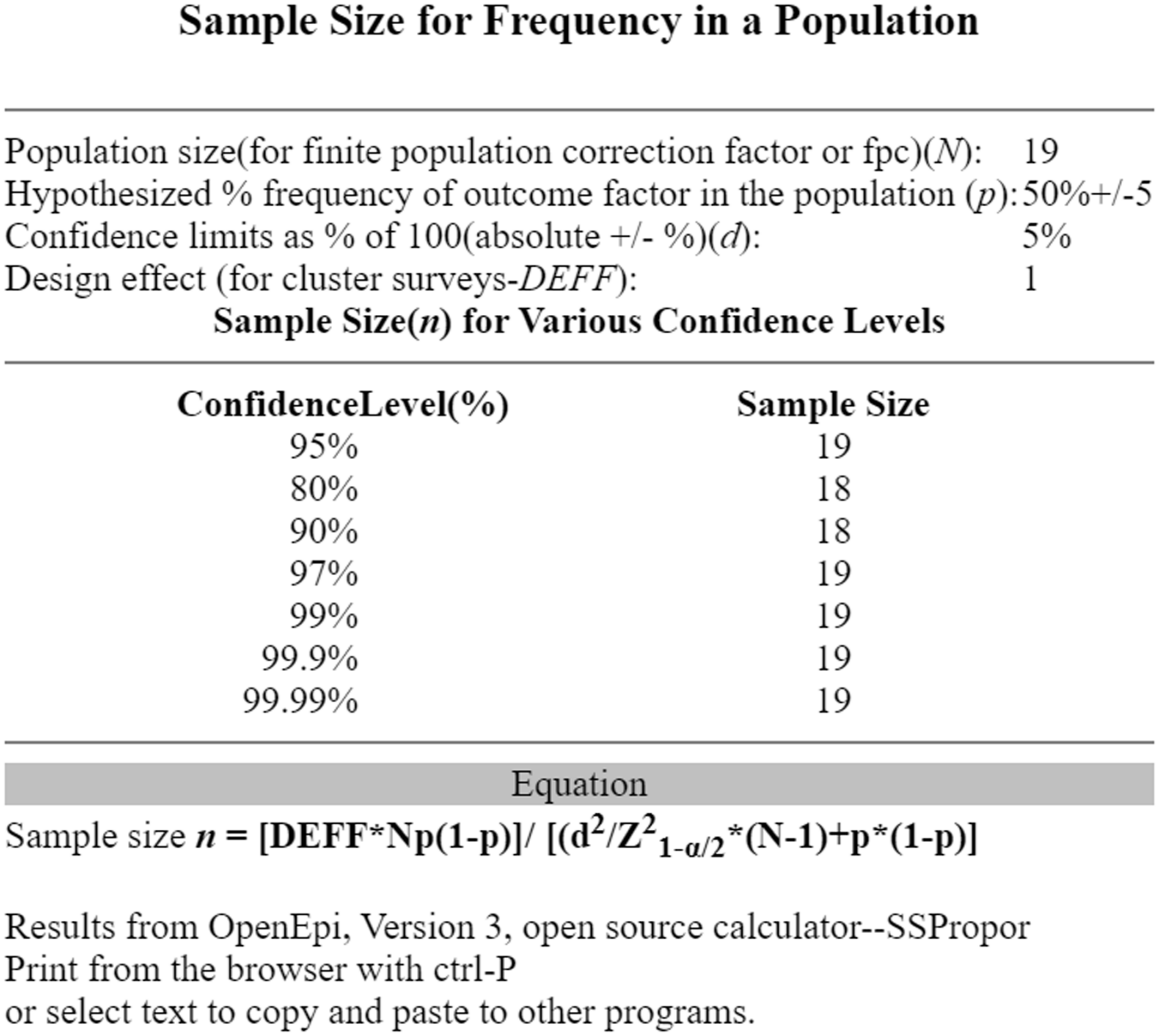
Sample size for frequency in the military pilot’s population using OpenEpi.

Within these criteria there are 19 individuals serving as military pilots actively engaged in their training (tirocinium) at the PAA, specifically within the PA course titled “Masters in Military Aeronautics: Aviator Pilot Specialist,” and our sample size is calculated with OpenEpi, with a 95% confidence interval, which corresponds to 19 subjects—the totality of this population, as seen in Image 1.

#### 3.2.2 Recruitment and screening

The collaboration between Évora University and the PA involved reaching out to the Head of the Chief of Staff Cabinet. Under this collaborative protocol, a Ph.D. student will administer a yoga intervention at the chosen airbase 11 in Beja, while a team from Évora University conducts on-site baseline and post-intervention data collection. The authorization for this collaborative effort was granted by the Portuguese Air Force’s Head of the Chief of Staff Cabinet.

Pilots will undergo a comprehensive briefing on the study’s general parameters and will be extended a voluntary invitation to participate. Following this briefing, participants will be required to sign an informed consent form in adherence to the Helsinki Declaration. Any queries or concerns can be addressed during this stage to ensure clarity and compliance with ethical standards.

#### 3.2.3 Randomization procedure and blinding

Upon inclusion, volunteers will be allocated to either a waiting list (control group) or a yoga class (intervention group) through a computer-based algorithm (random.org), to be facilitated by the team captain of the pilots. All study personnel, excluding the team captain and one study coordinator from Évora University, will be kept unaware of the group allocation. Participants will be explicitly instructed to refrain from disclosing their group assignment to the study team. To uphold impartiality, all data will be coded for subsequent processing and analysis, maintaining blindness to group allocation. The final coded trial dataset will be made available to all team members for analysis.

### 3.3 Experimental procedure

#### 3.3.1 Study flow

The study team will visit Airbase 5 in Monte Real to consult with the F16 Team Captain. The objectives include planning and scheduling visits, testing the proposed protocol, assessing all pertinent equipment, identifying, and rectifying potential errors before the initiation of the initial data collection. During this meeting, interviews with the Team Captain will be conducted to evaluate the perceived needs of pilots during their aircraft piloting duties. The insights obtained, combined with findings from the literature, will guide the tailoring of the yoga intervention.

A baseline data collection session will be organized in Airbase 11 in Beja, with study participants selected randomly based on availability and location undergoing evaluation over two consecutive days during pilots’ work hours from 8:30 to 19:00. Oversight of the data collection will be provided by experts in Sports Exercise and Health from Évora University, the study coordinator, and a simulator controller from the Air Force team.

The yoga group will integrate a one-hour yoga class into their work schedule, in Airbase 11 in Beja, twice a week for a 12-week period. For those on missions with conflicting schedules, alternative days and times will be accommodated. Each pilot is required to complete 24 yoga classes within the designated 12 weeks. The yoga intervention, led by the study coordinator—an Ashtanga Vinyasa Yoga expert with over 300 h of training and approximately 10 years of teaching experience—will be conducted.

Post-intervention data collection will follow the completion of the yoga program, with study participants, randomly divided based on availability and location, undergoing evaluation over two consecutive days, in Airbase 11 in Beja, during pilots’ work hours from 8:30 to 19:00. Supervision will be provided by experts in Sports Exercise and Health from Évora University, the study coordinator, and an available simulator controller from the Air Force team.

Upon the study’s conclusion, the waiting list group will be offered the same 12-week yoga classes before completing their tirocinium and departing the airbase, aligning with ethical considerations.

#### 3.3.2 Practical applicability

The communication strategy for disseminating trial results to participants and Air Force professionals involves presenting comprehensive data collection information, results, and conclusions to the Health and Exercise Department of the Portuguese Air Force. This initiative holds particular significance as there is currently no established formal exercise program for training aircraft pilots. The outcomes of this study may prove instrumental in the creation or enhancement of such a program, thereby contributing to the improvement of future military health policy.

For the broader public and other pertinent groups, access to the study’s findings can be facilitated through publication in scientific journals. This dissemination approach ensures that the study’s outcomes are accessible to a wider audience, contributing to the broader scientific and professional knowledge base.

#### 3.3.3 Arms and intervention

Table 1 outlines the study arms: the control group receiving military training alone and the intervention group undergoing both military training and yoga classes.

**Table 1:** Arms and assigned interventions of the study protocol.

Arms	Assigned interventions
Experimental: Yoga	Yoga practice: 12-week program of Ashtanga Vinyasa Yoga Supta for 1 h twice a week; other names – yoga class
No Intervention: Waiting list	Exclusively compulsory training (the same for all academy attendees).

**IMAGE 2:**
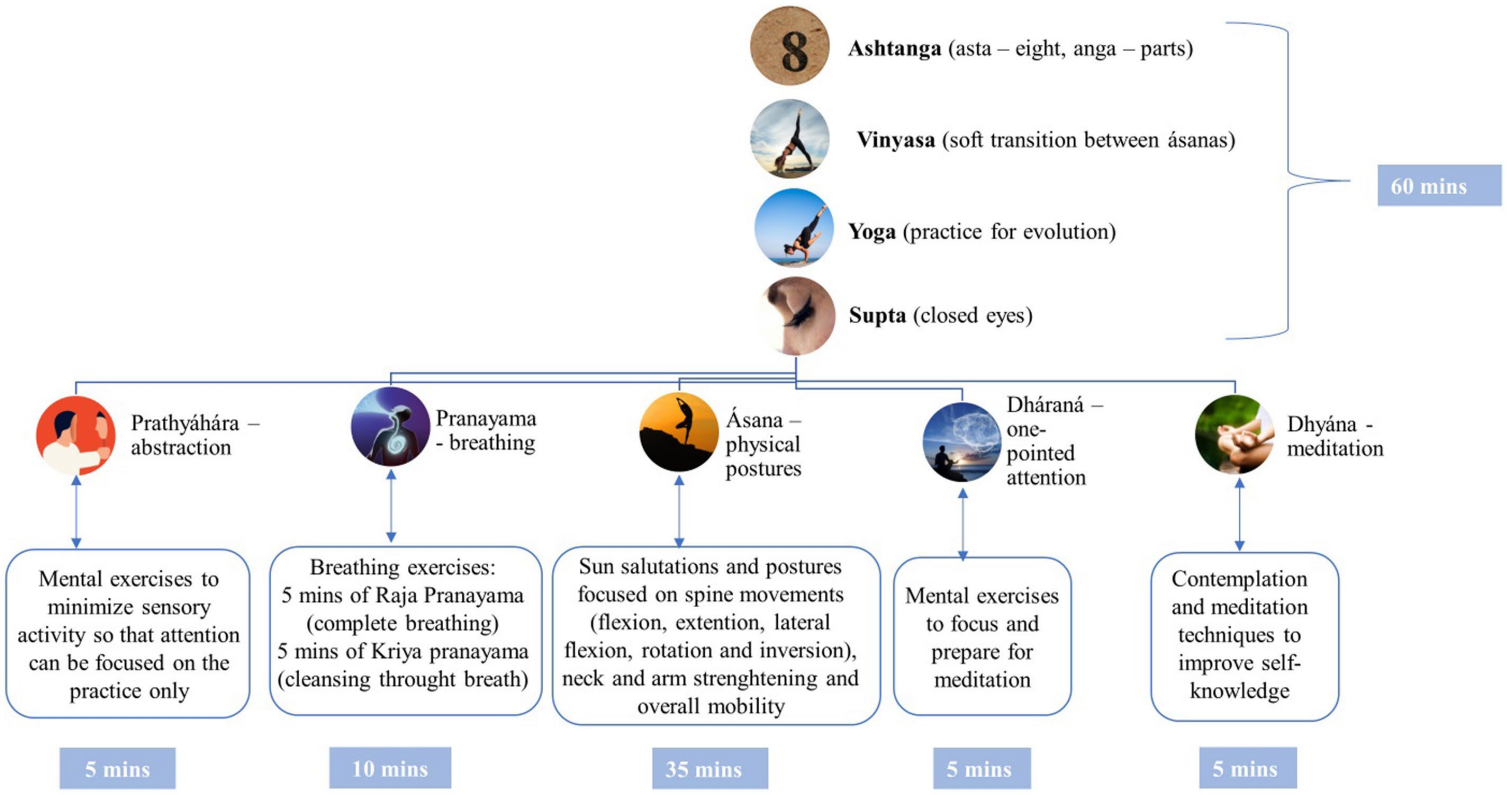
AVYSM classes structure and components.

**IMAGE 3:**
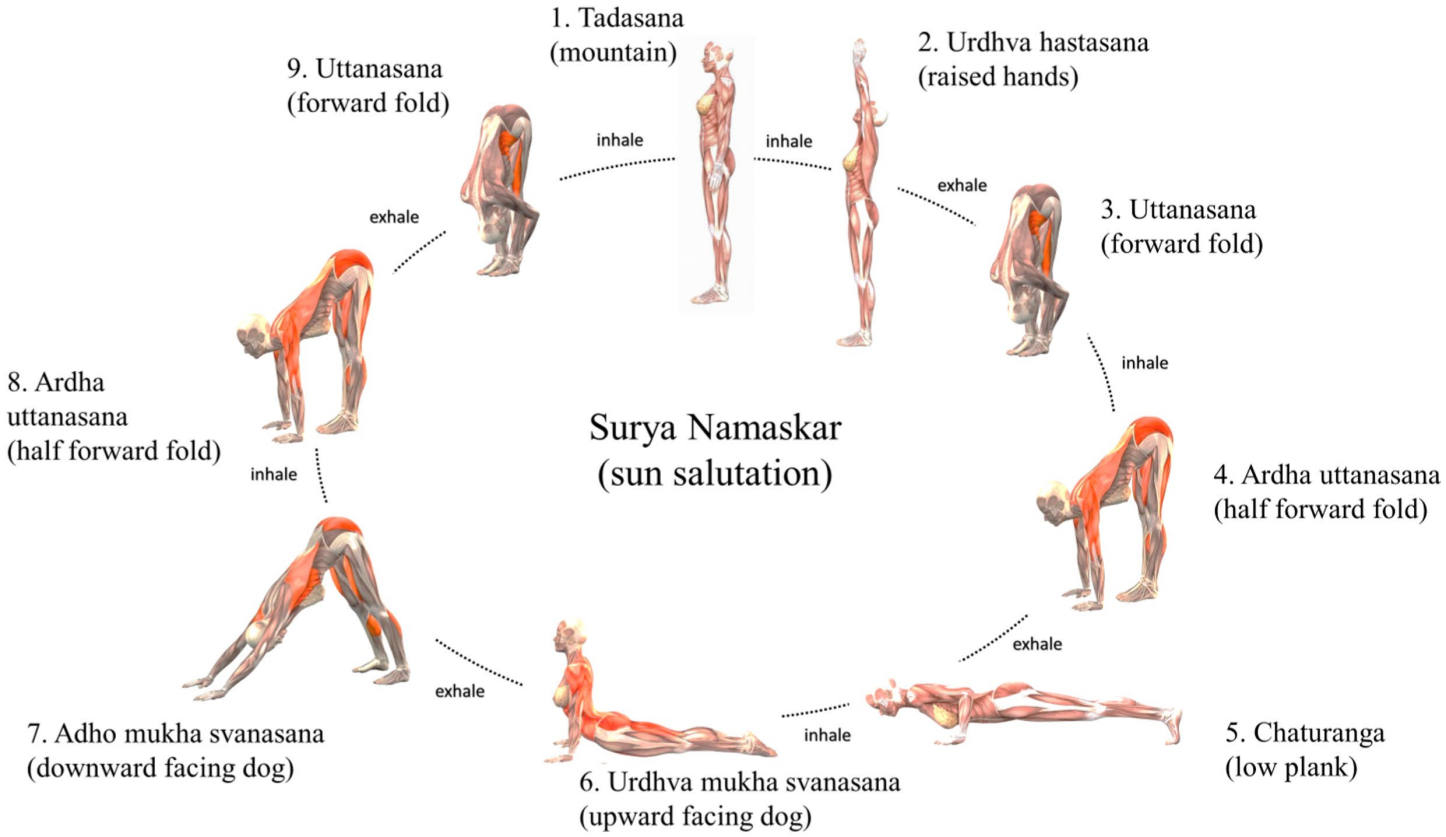
Sun salutations (28) used in the ásana component of the AVYSM classes.

**IMAGE 4:**
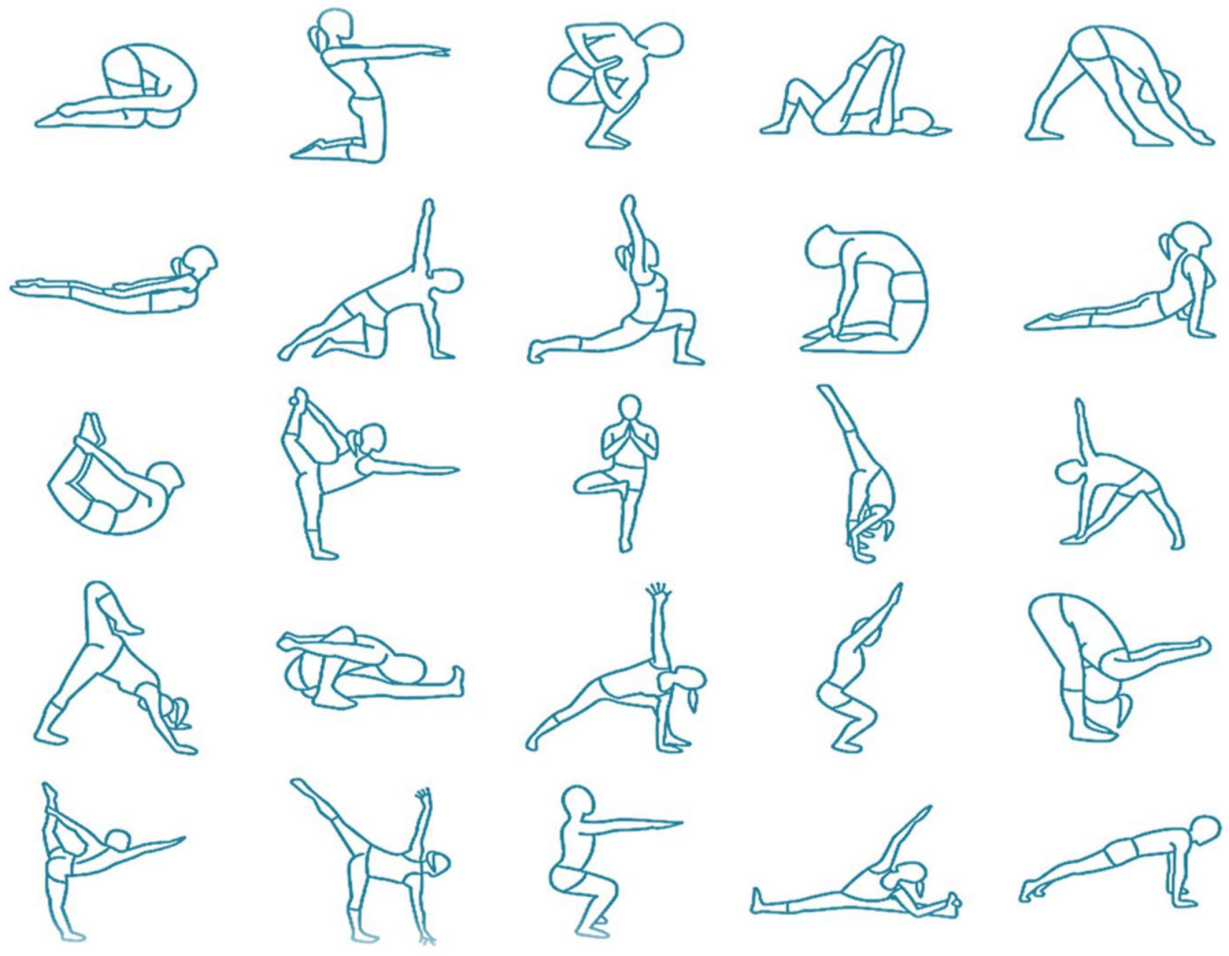
Various yoga postures (ásanas) incorporated in the ásana component of the AVYSM classes.

The yoga practice, as depicted in Image 2, entails fluid sequences (Ashtanga Vinyasa Yoga) performed with closed eyes (supta) to address perceived difficulties in pilots. The yoga classes comprise five main components:

- 5 min of prathyáhara (abstraction)—“withdrawal of the senses” or “sensory withdrawal,” involves consciously detaching the senses from external stimuli and directing attention inward. Through pratyahara, practitioners aim to disengage from external distractions, facilitating a deeper exploration of the inner self and enhancing self-awareness.
- 5 min of pranayama (breathwork, including raja pranayama and kryia)—these are advanced forms of breathwork aimed at regulating the breath and influencing the flow of prana (life force energy) throughout the body. Raja Pranayama focuses on controlling the breath through various breath retention methods, such as inhaling (puraka), holding the breath (kumbhaka), and exhaling (rechaka) in specific ratios. Kriya pranayama techniques typically combine specific breath control, body postures, and mental focus to influence the flow of prana (life force energy) throughout the body. Nauli Kriya is an advanced yogic purification technique that involves isolating and rhythmically churning the abdominal muscles. It is performed by contracting and rolling the abdominal muscles in a specific manner to create a rolling or rippling motion across the abdomen.
- 35 min of ásana (physical postures)—with sun salutations, as depicted in Image 3 and sequenced physical postures, as depicted in Image 4, involving spinal flexion, extension, lateral flexion, rotation, and inversions, changes in head positioning entailing a recalibration involving the integration of visual, vestibular, and muscular sensations, combined with breath control and abstraction, for a holistic approach to health and well-being.
- 5 min of dhāraṇā (practice of concentration or single-pointed focus)—beginning with yoga nidra for physiological relaxation, followed by mental exercises to focus and prepare for meditation. Yoga nidra is the practice of deep relaxation or yogic sleep. It is a state of consciousness between wakefulness and sleep, where the body and mind are in a state of profound rest while remaining fully aware. During yoga nidra, practitioners typically lie down in a comfortable position and follow guided instructions to systematically relax different parts of the body, release tension, and enter a state of deep relaxation. The practice often involves techniques such as body scanning, breath awareness, visualization, and mindfulness. Dhāraṇā involves directing one’s attention to a specific object, thought, or sensation, and maintaining that focus without distraction. This object of focus could be anything, such as a physical object, a mental image, a mantra, or the breath. The goal of dhāraṇā is to develop greater mental discipline, control over the wandering mind, and the ability to sustain attention for prolonged periods.
- 5 min of dhyána (meditation or contemplation)—involves focused concentration and sustained attention on an object, thought, or sensation to achieve a state of mental clarity, stillness, and inner awareness. During dhyana, practitioners aim to quiet the mind, transcend distractions, and cultivate a deep sense of presence and mindfulness, focused on developing greater mental resilience and emotional balance. Sankalpa (“resolve” or “intention”) was also used during meditation, it is a tool used to set a clear and positive intention for one’s life or practice, a heartfelt affirmation of one’s deepest values, aspirations, and purpose. It serves as a constant reminder of what truly matters and empowers individuals to align their thoughts, words, and deeds with their deepest intentions.

The components will be adapted to the specific needs of the pilot group during their flight missions: supta classes, primarily or entirely conducted with closed eyes, aim to eliminate visual system responses and evoke vestibular system responses; Ashtanga Vinyasa classes emphasize fluid movement sequences, focusing on breathing and enhancing neck and upper limb strength through various yogic movements available to the head and spine (flexion, extension, lateral flexion, rotation, and inversion). This recalibration facilitates the pilot’s awareness of their spatial orientation, complemented by improvements in embodiment and proprioception. This, in turn, enhances the pilot’s perception of the aircraft’s orientation and their own position in relation to it, as well as their spatial awareness of the aircraft in relation to the Earth. The classes will commence with basic-level techniques, progressing to more advanced ones as the intervention group’s proficiency and abilities improve over the timeline.

#### 3.3.4 Statistical analysis

Sample size was calculated with OpenEpi. Data analyses will be done with MATLAB (18) and Jamovi (29), based on the type of data; analyses will be performed based on the underlying assumptions for parametric, or non-parametric testing. In detail, for all difference testing, data and variance distribution (i.e., normality) will be checked. The alpha-error threshold is set at 5%, all *p* values below are considered significant.

For the primary outcomes related with postural control and heart rate variability a MATLAB (18) routine will be used to treat data exported from the Biosignals Plux platform and also from the Polar H10 portable monitor, and with those values a Shapiro–Wilk normality test will be performed and either a T-test for paired samples will be applied on parametric data or a Wilcoxon rank test will be applied on non-parametric data.

For the primary outcomes related with operative performance and the secondary outcomes a Shapiro–Wilk normality test will be performed and either a T-test for paired samples will be applied on parametric data or a Wilcoxon rank test will be applied on non-parametric data.

## 4 Results

The principal objective of the study is to assess the feasibility and efficacy of implementing a 12-week yoga program to enhance existing military training protocols within the Portuguese Air Force. This will be accomplished through the utilization of a randomized controlled intervention design. The intervention comprises preventive exercises tailored to address perceived challenges faced by pilots during aircraft piloting, thereby enhancing the capabilities of individuals who are already undergoing training.

The findings of this study hold significance for the research group and are equally pertinent for policymakers within the Portuguese Air Force. The data gathered in this project can aid policy makers in developing a tailored exercise regimen.

Broadening the study’s relevance to diverse contexts, the methods, results, and discussions generated by this project have the potential to be extrapolated to diverse military contexts, beyond military aviation, exploring applications of human intervention in different military settings, such as incorporating new training techniques for: airfield operations officers, paratroopers, armored vehicle operators, tank crews, navy divers, military paratroopers, and others, where the improvement of physical and cognitive abilities aligns with the outcomes of this study. Furthermore, the applicability of the project’s findings extends to high-demand environments beyond military, including civil aviation (both for pilots and air traffic controllers), professional race car or motorcycle drivers and skydiving companies—professions where the optimization of physical and cognitive capabilities may offer valuable insights and benefits.

## 5 Discussion

Recognizing the critical role of cognitive performance in military operations, where impaired cognitive function is a prominent factor in accidents during training and combat (30), instructors must prioritize not only the physical health but also the psychological well-being of trainee pilots (31). The potential to enhance sensory systems, particularly through vestibular habituation and adaptation, can influence sensory weighting and postural behavior, the processing of sensations, along with their identification and integration with others, providing a comprehensive framework of possibilities for decision-making and action planning (32). Consistent yoga practice contributes to ongoing improvement in postural control, muscle strength, and the vestibular system, fostering increased plasticity in the sacculocolic pathway and resulting in enhanced vestibular evoked myogenic potential (cVEMP) responses (32). Furthermore, Ashtanga Yoga, in accordance with Patanjali, encompasses techniques that address all facets of the human system, including the body, breath, mind, personality, and emotions (11).

A notable strength of this intervention lies in its practicality and cost-effectiveness. Furthermore, all exercises are executable without the need for additional materials and can be conducted in spaces already designated for training, such as a gym room or outdoors when weather conditions permit.

The sample size may present challenges in interpreting results and deducing the practical relevance of the study. Challenges in maintaining blinding among pilots also present potential limitations. These are mitigated by including the totality of this population, designating the control group as a waiting list, offering them the same intervention subsequently, and ensuring blinding for all investigators except the study coordinator.

As civilians, we secured explicit authorization to conduct this study within a military setting. Compliance with specific military regulations, legal constraints, and adherence to classified information protocols were paramount. Notably, due to operational constraints, especially in classified, restricted or warzone areas, the team could only study and publish information from active pilots taking their Tirocinium. The team was able to study the pilots while they worked, gaining hands-on experience and practical skills before assuming their full duties. Testing moments had to synchronize with the pilots’ professional commitments and occur in non-classified areas. The study focused solely on Tirocinium Pilots, given the limitations in examining Airforce Pilots. These measures ensured alignment with legal, security, and operational considerations during the study.

Currently, the Portuguese Air Force’s Health and Exercise Department lacks an official exercise training program for pilots. Pilots are generally instructed to train in a manner they deem suitable to meet mandatory physical testing requirements. Information obtained from this study holds potential utility in the development of a structured training program and the adaptation of physical evaluation tools, thereby improving future military health policy and legislation. This research’s potential extends beyond the PA, as it can be adapted for use in Airforce departments of other nations and various military or high-performance contexts.

## Data availability statement

The original contributions presented in the study are included in the article/supplementary material, further inquiries can be directed to the corresponding author.

## Ethics statement

The studies involving humans were approved by Évora University research ethics committee—approval number 21050. The studies were conducted in accordance with the local legislation and institutional requirements. The participants provided their written informed consent to participate in this study.

## Author contributions

SS: Conceptualization, Data curation, Investigation, Methodology, Project administration, Resources, Software, Writing – original draft. FM: Conceptualization, Data curation, Formal analysis, Investigation, Methodology, Resources, Software, Supervision, Validation, Visualization, Writing – review & editing. OF: Conceptualization, Data curation, Formal analysis, Investigation, Methodology, Resources, Software, Supervision, Validation, Visualization, Writing – review & editing. JP: Conceptualization, Data curation, Formal analysis, Funding acquisition, Investigation, Methodology, Resources, Software, Supervision, Validation, Visualization, Writing – review & editing.
